# Inflammatory and Immune Mechanisms for Atherosclerotic Cardiovascular Disease in HIV

**DOI:** 10.3390/ijms25137266

**Published:** 2024-07-01

**Authors:** Laura Hmiel, Suyu Zhang, Laventa M. Obare, Marcela Araujo de Oliveira Santana, Celestine N. Wanjalla, Boghuma K. Titanji, Corrilynn O. Hileman, Shashwatee Bagchi

**Affiliations:** 1 Department of Medicine, Division of Infectious Disease, MetroHealth Medical Center and Case Western Reserve University, Cleveland, OH 44109, USA; 2 Department of Medicine, Emory University, Atlanta, GA 30322, USA; 3 Division of Infectious Diseases, Vanderbilt University Medical Center, Nashville, TN 37232, USA; 4 Department of Medicine, Washington University in St. Louis, St. Louis, MO 63110, USA; 5 Division of Infectious Diseases, Emory University, Atlanta, GA 30322, USA; 6 Division of Infectious Diseases, Washington University in St. Louis, St. Louis, MO 63110, USA

**Keywords:** atherosclerosis, HIV, inflammation, immune system, innate immune system, adaptive immune system, mechanisms, monocytes/macrophages, T lymphocytes, cardiovascular disease

## Abstract

Atherosclerotic vascular disease disproportionately affects persons living with HIV (PLWH) compared to those without. The reasons for the excess risk include dysregulated immune response and inflammation related to HIV infection itself, comorbid conditions, and co-infections. Here, we review an updated understanding of immune and inflammatory pathways underlying atherosclerosis in PLWH, including effects of viral products, soluble mediators and chemokines, innate and adaptive immune cells, and important co-infections. We also present potential therapeutic targets which may reduce cardiovascular risk in PLWH.

## 1. Introduction

Atherosclerotic cardiovascular disease (ASCVD) is an important cause of morbidity and mortality in persons living with human immunodeficiency virus (HIV) (PLWH), even when adjusting for known traditional risk factors [1]. The global burden of HIV-related cardiovascular disease (CVD) has tripled since the early 1990s, and PLWH are over twice as likely to develop CVD or myocardial infarction (MI) compared to people without HIV (PWoH) [2,3,4,5].

The reason for this heightened risk is multifactorial. While traditional CVD risk factors affect PLWH as well as PWoH, PLWH are more likely to be exposed to unique CVD risk factors. Prior reviews have extensively focused on CVD risk due to antiretroviral therapy (ART), especially older protease inhibitors such as lopinavir and ritonavir and the nucleoside reverse transcriptase inhibitor abacavir [6,7].

Inflammation and immune factors have been described in the pathogenesis of ASCVD in PWoH [8,9,10,11]. The interplay of inflammatory and immune pathways and HIV infection has been implicated as a possible explanation for the excess CVD risk in PLWH, and reviewed previously [12,13]. Moreover, the inflammatory response to HIV appears to be affected by co-infections, particularly viral infections like herpesviruses and hepatitis C virus (HCV) [14,15].

**Figure 1:**
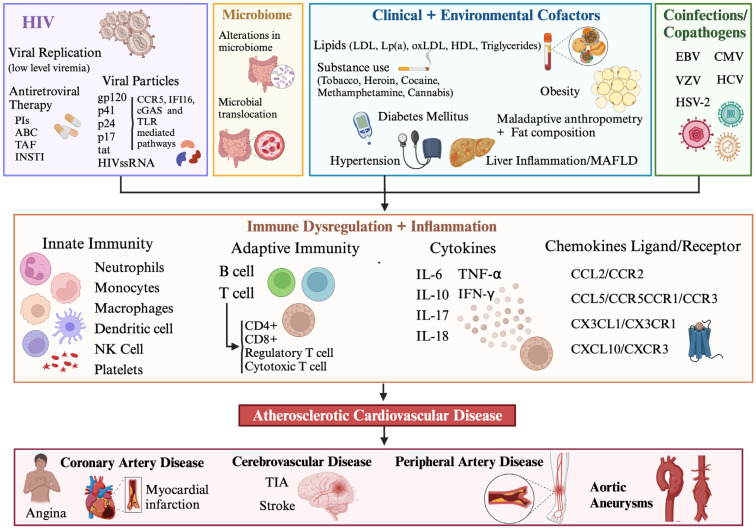
HIV-associated atherosclerosis results from a culmination of numerous intersecting pathways. HIV-related factors include viral proteins, viral ssRNA, viral replication, antiretroviral therapy, and the microbiome. Clinical and environmental co-factors include hypertension, dyslipidemia, diabetes mellitus, obesity, maladaptive anthropometry and fat composition, liver inflammation/metabolic dysfunction-associated fatty liver disease (MAFLD), and concurrent substance use. Important co-infections and co-pathogens include cytomegalovirus (CMV), varicella zoster virus (VZV), herpes simplex virus 2 (HSV-2), Epstein–Barr Virus (EBV), and hepatitis C virus (HCV). These HIV-related, clinical, and environmental co-factors and co-infections induce inflammation and immune dysfunction. Perturbations of cytokines, chemokines, and the innate and adaptive immune systems together result in immune dysregulation and inflammation, which lead to athrosclerosis and clinical atherosclerotic cardiovascular disease, cerebrovascular disease, peripheral artery disease, and aortic aneurysms. Abbreviations; ABC = abacavir; CCL = chemokine ligand; CCR = chemokine receptor; CXCL = CXC motif chemokine ligand; CXCR = CXC motif chemokine receptor; CX3CL = C-X3-C motif chemokine ligand; CX3CR = C-X3-C motif chemokine receptor; gp = glycoprotein; cGAS = cyclic GMP-AMP synthetase; HDL = high-density lipoprotein; IFI16 = interferon inducible protein 16; IFN = interferon; IL = interleukin; INSTI = integrase strand transfer inhibitor; LDL = low-density lipoprotein; Lp(a) = lipoprotein (a); LPS = lipopolysaccharide; MAFLD= metabolic-associated fatty liver disease; NK = natural killer; oxLDL = oxidized low-density lipoprotein; PIs = protease inhibitors; ssRNA = single-stranded ribonucleic acid; TAF = tenofovir alafenamide; tat = trans-Activator of transcription; TIA = transient ischemic attack; TLR = toll-like receptorl TNF = tumor necrosis factor.

In this review, we provide an updated review of the inflammatory and immune mechanisms that contribute to ASCVD in PLWH, with a focus on the innate and adaptive immune mediators of atherosclerosis and the effects of co-pathogens such as HCV, cytomegalovirus (CMV), herpes simplex virus (HSV), and varicella zoster virus (VZV) on the mechanisms of ASCVD in HIV (Figure 1).

## 2. Regulation of Inflammation in PLWH

### 2.1. HIV-Related: Viral Replication, Viral Reservoir, Viral Proteins

Early in the HIV epidemic, depletion of CD4^+^ T cells was a major predictor of morbidity and mortality in PLWH. As ART improved, it became evident that risk of death was not explained by CD4^+^ T cell count alone but also depended on duration of infection and viremia [16]. HIV itself leads to a dysregulated immune response to bacterial and viral markers including lipopolysaccharide (LPS), resiquimod (R848), and polyinosinic:polycytidylic acid (Poly I:C) [17] while simultaneously causing intrinsic activation by HIV proteins. Furthermore, despite ART-mediated suppression of plasma HIV RNA, the virus persists in reservoirs where continued low-level production of viral proteins and RNA leads to production of pathogen-associated molecular patterns (PAMPs) and activation of pattern recognition receptors (PRR) like toll-like receptors (TLR), which promote pro-inflammatory cytokine signaling [18]. For example, HIV-1 gp120 induces interleukin-1β (IL-1β) release from macrophages through chemokine receptor 5 (CCR5)-mediated signaling pathways, and other proteins including p17, p24, and p41, all functioning as PAMPs that activate CCR5- and/or TLR-mediated pathways [19,20]. Other PRRs of note include retinoic acid-inducible gene I (RIG-I), cyclic GMP-AMP synthetase (cGAS), and interferon inducible protein 16 (IFI16). IFI16 and cGAS both recognize complementary HIV DNA and trigger pro-inflammatory stimulation of interferon genes (*STING*)-mediated cascades through transcription of the *IFN-I* gene, NF-κB pathways, and inflammasome activation with pyroptosis of affected T cells [21,22]. Levels of *STING* and *cGAS* gene expression subsequently decrease with viral suppression on ART [23]. HIV ssRNA itself activates multiple endosomal TLRs which promote continued inflammation, including TLR7-mediated plasmacytoid dendritic cell activation and TLR8-mediated T cell activation and differentiation to Th1/17 cells and HIV reactivation [24,25]. Furthermore, certain accessory HIV-1 proteins like Vpu and Vpr can modulate the immune response to infection in multiple ways, including downregulating interferon regulatory transcription factor 3 (*IRF3)* gene expression and subsequent interferon-based pathways, potentially allowing viral evasion of the innate immune system [21,26].

### 2.2. Alterations of the Microbiome

Gut-mucosal-associated T cell populations represent a significant portion of the lymphoid cells infected by HIV, and the gut mucosa becomes a primary location for viral replication even while on ART [27]. Microbial translocation occurs through epithelial cell damage resulting in systemic inflammation and immune dysregulation [28]. Higher HIV viral loads and lower CD4^+^ T cell counts correlate with elevated plasma concentrations of bacterial LPS, as well as increased host IL-6, interferon-α (IFN-α), tumor necrosis factor-α (TNF-α), and monocyte and T cell activation [28,29,30,31]. Beta-D-glucan, a fungal cell wall product, can also be elevated in PLWH and is associated with elevated TNF-α and IL-8, epithelial cell damage, and even directly with coronary atherosclerotic plaque [32,33].

HIV infection also affects the microbiome, leading to decreased microbial diversity and shifts in composition, with greater effects at lower CD4^+^ T cell counts [31]. In high-income western countries, this altered microbiome becomes enriched in opportunistic bacteria including Prevotella and Proteobacteria and diminished in more commensal protective bacteria such as Bacteroides [34]. This shift in microbiome is associated with enhanced local and systemic T cell and dendritic cell responses, as well as rise in pro-inflammatory cytokines associated with mortality including IL-6, TNF-α, IL-1β, and interferon-γ (IFN-γ) [35]. The same shift also leads to a rise in signaling molecules like soluble CD14 (sCD14), which binds LPS in the presence of LPS-binding protein and subsequently activates TLR-4 on myeloid cells [36,37]. While HIV affects the specific microbiome footprint, geography and sexual orientation have robust impacts as well [36].

### 2.3. Lipids

Dyslipidemia in PLWH typically presents as hypocholesterolemia with relatively lower high-density lipoprotein cholesterol (HDL-c) and higher triglyceride levels than controls [38,39,40], although some studies note lower or similar levels of triglycerides [41]. Sufficiently high HDL-c concentration and function are crucial for cholesterol clearance and are generally anti-atherogenic [42]. However, altered lipoprotein particles and changes in particle sizes (e.g., smaller HDL particles) may correlate better with overall cardiovascular risk in PLWH [43,44]. Lipoprotein (a) is a subset of low-density lipoprotein (LDL) that has been associated with CVD risk and inflammatory markers in PWoH. It has similarly been associated with increased systemic inflammatory markers like IL-6, high-sensitivity C-reactive protein (hs-CRP), and soluble TNFR-I and -II; markers of monocyte activation; and peri-coronary inflammation in PLWH [45]. Additionally, oxidized LDL (oxLDL), which is associated with surrogate measures of atherosclerosis, is present in higher concentrations in PLWH and induces atherogenic pathways as measured by increased sCD14 levels and monocyte activation [46,47].

HDL-mediated reverse cholesterol transport from macrophages to the liver is crucial for prevention of atherogenesis and is impaired in PLWH. HIV production of Nef protein, which is crucial to persistence of HIV infection, disrupts this process and promotes accumulation of cholesterol in macrophages, resulting in foam cells implicated in fatty streak formation [48,49]. Additionally, monocytes in PLWH exhibit lower gene expression of ATP binding cassette subfamily A member 1 (*ABCA1*), which is transcribed to an ATP-binding cassette transporter crucial for reverse cholesterol transcription [50]. These alterations in macrophage and monocyte function are discussed further in the section on innate immunity.

### 2.4. Substance Use: Heroin, Cocaine, Methamphetamine, Tobacco, Cannabis

Many PLWH have concomitant substance use disorders, with opioids use disorder (OUD) representing a major public health crisis. Aortic inflammation, which correlates with CVD and atherosclerosis, was found to be increased in PLWH who use heroin compared to both PWoH and PLWH who do not use heroin[51]. Heroin use similarly tends to be associated with markers of microbial translocation and systemic inflammation in PLWH, although this may be independent of HIV status [52]. PLWH who use opioids have demonstrated blunted innate immune responses of monocytes and T cells compared to PLWH without OUD, with reduced production of IL-10, IL-8, IL-6, IL-1α, and TNF-α when exposed to LPS [53].

Stimulants including cocaine and methamphetamines carry well-known cardiovascular risks. Methamphetamine use, especially intravenously, correlates with higher levels of inflammatory markers hs-CRP, IL-6, sTNFR-1, immune activation markers sCD163 and sCD14, and the microbial translocation marker LPS binding protein [54,55]. Similar inflammatory and immune-activating markers including hs-CRP and sCD14 are increased in PLWH who use stimulants such as cocaine or methamphetamines [55].

Tobacco smoking is disproportionately prevalent in PLWH, with between 40 and 70% estimated to smoke tobacco, and has an outsized effect on atherogenesis compared to PWoH [56,57]. Tobacco smoke impairs innate immune cell responses to pathogens while also upregulating activation of growth factors and cytokines including IL-6, IL-8, and TNF-α, promoting high-risk coronary plaque development in PLWH [57,58].

Cannabis use is common among PLWH and has overall anti-inflammatory effects through endogenous cannabinoid pathways, down-regulating inflammatory cell lines like activated T cells and improving gut barrier integrity [59,60]. However, cannabis use is associated with cardiovascular disease including stroke, even when adjusting for confounders such as tobacco smoking [60,61]. Further research into the inflammatory and immune mechanisms of cannabis on vascular health may elucidate the overall effect of cannabis on cardiovascular risk and inflammation after balancing various pathways.

## 3. Soluble Mediators

The human immune system comprises both cellular components as well as cytokines and other soluble mediators. Cytokines and soluble mediators play integral roles in regulating and driving the inflammation underlying atherosclerosis through downstream autocrine, paracrine, and endocrine effects. Of these, interleukins are critical mediators of HIV-associated atherosclerosis. Broadly characterized as pro- and anti-inflammatory, these cytokines along with other soluble mediators play a direct role in shaping the inflammatory and immune response through the innate and adaptive immune systems [12,62,63,64]. The effects of key systemic inflammatory mediators on atherosclerosis are summarized below.

ART-suppressed PLWH exhibit significantly higher levels of pro-inflammatory cytokines such as IL-1β, IL-6, and TNF-α compared to PWoH [65]. Soluble mediators IL-6, TNF-α, and D-dimer are associated with chronic HIV infection as well as various drivers of atherosclerosis including endothelial dysfunction, increased carotid intima-media thickness (cIMT), and transmigration of inflammatory subsets of monocytes [12,66,67,68,69]. Elevated levels of IL-6 and D-dimer have also been associated with greater risk of major adverse cardiac events (MACE) and CVD in PLWH [70,71,72,73,74,75]. In a study examining the effect of ART initiation on inflammation, women with HIV had persistently higher levels of IL-6, IL-2 receptors, and D-dimer, and had higher levels of subclinical atherosclerosis as measured by cIMT compared to women without HIV [68]. As in PWoH, hs-CRP correlates not only with general inflammation but also CVD, including type 1 MI, and overall mortality after CVD events [70,76]. However, hs-CRP has been variably predictive of CVD and all-cause mortality [71,77].

IL-10 is typically an anti-inflammatory cytokine associated with decreased plaque burden in PLWH [62,78,79]. In some studies, levels have been found to be comparable in PLWH and controls despite decreased plaque burden, which suggests that IL-10 function in the setting of HIV infection may have pronounced effects in local microenvironment, possibly mediated by differential milieu of immune cells or higher local tissue concentrations [62,78,79]. Such discordance of differential effects of plasma or serum levels with tissue level or clinical outcomes has been observed with other biomarkers previously as well [45,77,80].

IL-17a is a pro-inflammatory cytokine primarily produced by Th17 cells, which typically modulates inflammation through its effect on chemokine expression and leukocyte migration [81]. HIV infection depletes Th17 cells, and subsequently promotes the atherosclerotic kynurenine tryptophan catabolic pathway as measured by kynurenine–tryptophan ratio, both of which incompletely normalize after initiation of ART [82,83,84]. IL-17a additionally may promote atherogenesis through its effect on monocytes and macrophages [85]. In PLWH, IL-17a has been shown to be associated with endothelial dysfunction and is theorized as an important driver of inflammation leading to atherosclerosis [82]. 

IL-18 is another pro-inflammatory cytokine which has been demonstrated to be upregulated in PLWH and has been associated with subclinical atherosclerosis [86]. IL-18 is associated with monocyte and in vitro monocyte-derived macrophage (MDM)-induced inflammasome inflammation [87].

Chemokines and chemokine receptors are integral players in inflammation and inflammatory conditions [88]. Following interaction with their specific chemokine ligands, chemokine receptors trigger a flux in intracellular calcium ions that leads to cellular responses like the onset of chemotaxis of cells to a specific location within the body. Atherosclerosis involves the engagement of chemokines on the surface of the vascular endothelium and the recruitment of inflammatory cells into the vessel wall [89]. CCR5 is one important co-receptor for macrophage-tropic (M) and dual (M and T cell)-tropic HIV-1 [90], and C-X3-C motif chemokine receptor 1 (CX3CR1) is a minor co-receptor for HIV-1 [91]. CCR5^+^CD4^+^ T cells are upregulated in PLWH compared to PWoH, and higher proportions of CX3CR1^+^ CD4^+^ and CD8^+^ T cells have been noted in HIV [92,93]. Increased CCR2 expression on monocytes has also been observed in HIV [94]. Finally, expression of CXC motif chemokine ligand (CXCL10), a ligand which binds the chemokine receptor CXCR3, is increased in PLWH [28,95]. Among PLWH on stable ART, increased CCR5^+^CD8^+^ T cells, CCR5^+^CD4^+^ naïve and effector T cells, and monocyte markers were observed at least one year prior to first acute coronary syndrome (ACS) compared to matched controls who had not experienced ACS [96]. Also in ART-suppressed PLWH, CD16^+^ monocytes expressing CX3CR1 independently predicted cIMT [97], and higher frequency of CX3CR1^+^CD4^+^ T cells was associated with higher baseline and progression of cIMT regardless of HIV viral load or protease inhibitor use [98]. Elevated CXCL10 levels are known to be associated with increased ASCVD risk [99], and these levels return towards normal in PLWH virally suppressed on ART [28,95]. These observations suggest that CCR5, CX3CR1, CXCL10, and perhaps also CCR2 may have heightened significance in modulating atherosclerosis in HIV.

Table 1 lists important available or promising therapeutic agents targeting these soluble mediators of inflammation in atherosclerosis, as well as those targeting innate and adaptive immune cells discussed later.

## 4. Innate Immunity

**Figure 2:**
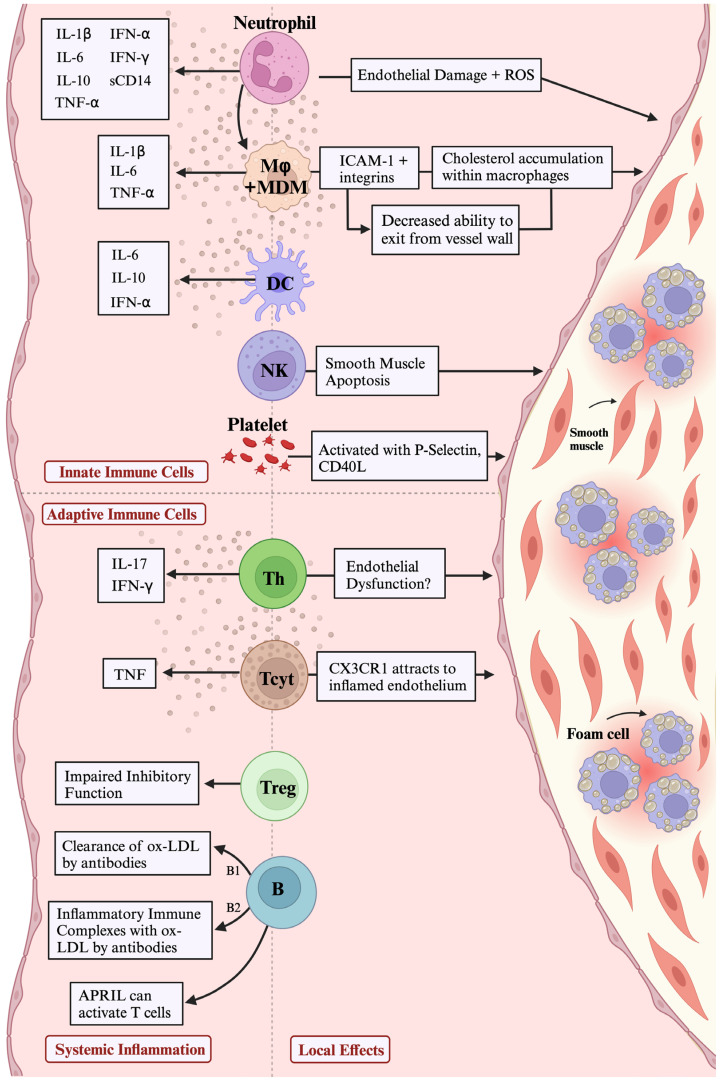
Innate and adaptive immune cells contribute in multiple ways to atherosclerosis via inflammatory pathways. The left side of the figure shows soluble mediators released by innate and adaptive immune cells that contribute to vascular inflammation. The right side of the figure shows how cells are drawn to, interact with, and damage blood vessel walls directly and contribute to plaque formation and destabilization. Abbreviations: APRIL = a proliferation-inducing ligand; B = B cell, including B1 and B2 subtypes; CD = cluster of differentiation; CX3CR= C-X3-C motif chemokine receptor; DC = dendritic cell; ICAM = intercellular adhesion molecule; IFN= interferon; IL= interleukin; Mφ = macrophage; MDM = monocyte-derived macrophage; NK = natural killer cell; ox-LDL= oxidized low-density lipoprotein; ROS = reactive oxygen species; Tcyt = cytotoxic T cell, including CD4^+^ and CD8^+^ subtypes; Th = T helper cell, including Th1, Th2, Th9, and Th17 subtypes; Treg = regulatory T cell.

The innate immune system plays an integral role in the initiation, establishment, and progression of ASCVD (Figure 2) [143]. Among a growing list of cells under investigation, special attention is paid to neutrophils, monocytes and macrophages, and dendritic cells (DCs) for their significant roles in inflammation and subsequent atherogenesis [10]. These innate immune cells and their dysregulation play critical roles in HIV pathogenesis and are increasingly recognized as having significant roles in HIV-mediated atherogenesis and ASCVD [144,145]. The mechanisms through which innate immune activation and dysregulation contribute to inflammation and subsequent atherogenesis in HIV infection are summarized below.

### 4.1. Neutrophils

Neutrophils are critical to the development of atherosclerosis in PWoH [146,147]. In PLWH, the role of neutrophil dysfunction in accelerating atherosclerosis has become a significant focus of research [148]. Chronic HIV infection results in elevated IL-8 levels and the expression of various integrins which upregulate and activate neutrophils. These activated neutrophils activate monocytes and MDMs through surface markers like CD11b/CD18 and CD11c/CD18 [148]. Moreover, neutrophils in PLWH both on and off ART display diminished chemotaxis, phagocytosis, bactericidal activity, and oxidative capacity during active viral infection. Concurrently, the dysregulated oxidative metabolism of neutrophils results in higher-than-normal reactive oxygen species (ROS) and oxidative stress, likely contributing to endothelial damage and intracellular stress [148,149,150,151]. This impairment in routine immune functions may contribute to chronic inflammation and endothelial damage caused by foreign pathogens. Finally, the role of neutrophils in gastrointestinal mucosal dysfunction and gut microbial translocation are currently being studied as independent factors in chronic inflammation and atherosclerosis [148]. Therapeutic approaches targeting neutrophil dysfunction in HIV-mediated atherosclerosis have focused on various aspects of neutrophil function, including more recently on the modulation of neutrophil extracellular traps as a therapeutic target for coronary artery disease.

### 4.2. Monocytes and Macrophages

In HIV infection, monocytes and macrophages contribute to atherosclerosis through altered innate immune signaling which activates a pro-inflammatory phenotype in monocytes and subsequently in macrophages. Chronic inflammation induces endothelial dysfunction, while monocyte and macrophage dysfunction can result in alterations in lipid processing and metabolism [122].

Several studies have shown that PLWH have elevated markers of myeloid cell activation [152]. Pro-inflammatory monocytes and MDM subsets (CD14^+^CD16^+^, CD38^+^, CD69^+^, CB11b^+^, sCD163^+^) in PLWH are linked to the development of carotid artery plaque [139,152,153,154,155]. The mechanisms through which HIV activates pro-inflammatory subsets of monocytes and MDMs are not fully elucidated but may be the result of direct HIV infection and circulating viral proteins [156]. Systemic inflammation with release of inflammatory cytokines and acute phase proteins, as well as chronic endotoxemia associated with HIV infection-mediated gut microbial translocation, also contribute to activation of myeloid cell subsets [153]. Monocyte function declines when ART is interrupted, causing HIV viremia. This suggests that in well-controlled HIV infection, monocytes and macrophages are not fully restored to normal but remain in a state of immune dysregulation [65].

Differential gene expression analysis of MDMs from PLWH has provided novel insights into the characteristics of monocytes and MDMs. A recent study examining peripheral blood monocytes identified extensive changes in innate immune signaling, and the expression of cell markers and immune receptors such as CD163, TLR4, and CD300e in monocytes and MDMs from PLWH [122]. These cells produced higher levels of inflammatory molecules associated with atherosclerosis, including TNF-α, ROS, and matrix metalloproteinases, in PLWH compared with cells in PWoH [122].

HIV infection causes endothelial dysfunction and alters monocyte migration and adhesion. This, combined with disrupted macrophage differentiation and lipid metabolism, leads to cholesterol accumulation in MDMs, forming foam cells and atheromas [153]. HIV infection can also directly stimulate increased expression of cell adhesion molecules such as CD11/CD18 integrins and intracellular adhesion molecule-1 (ICAM-1), which promote monocyte endothelial adhesion and extravascular dissemination [153,157]. Fibronectin fragments, created by proteases activated under inflammatory conditions, are present at high levels in PLWH and facilitate the trans-endothelial migration of HIV-infected mononuclear cells [158]. Additionally, research indicates that HIV-1 infection of macrophages impairs their ability to perform reverse trans-endothelial migration, a critical component of mononuclear cell immune surveillance and patrolling. This impairment may contribute to the formation of viral reservoirs, macrophage dysfunction, and the development of foam cells and chronic inflammation sites [50,159].

HIV-mediated macrophage dysfunction is a crucial area of focus for understanding HIV-associated atherosclerosis. Cellular and genomic studies have shown that macrophages from PLWH exhibit significant changes in innate immune signaling, genetic transcriptional modifications, and altered lipid processing [122,160,161]. HIV infection and associated viral proteins disrupt cholesterol efflux from macrophages, notably through downregulation of a membrane transporter protein called ABCA1 that is critical for cholesterol efflux and prevents excessive buildup of cholesterol within macrophages, which would lead to foam cell formation and atherosclerosis [153,160]. Chronic HIV infection and inflammation with increased levels of IL-6 downregulates lipoprotein lipase activity and increases macrophage uptake of lipids, further disrupting normal lipid metabolism and immunological homeostasis [162]. Additionally, an in vitro study demonstrated that HIV ssRNAs induced foam cell formation in MDMs in a dose-dependent manner, with increased TNF-α release triggered by TLR8 activation, suggesting a potential target for therapeutic intervention [163]. Current investigations of therapeutic strategies targeting macrophage dysfunction in HIV-induced atherosclerosis involve efforts to target and downregulate dysfunctional monocyte and macrophage recruitment. 

### 4.3. Dendritic Cells

DCs represent another important subset of the innate immune system, as they have multiple roles in facilitating atherosclerosis [164,165]. DCs play an active role in recruitment and activation of the adaptive immune system and also directly transmit HIV to CD4^+^ T cells. Furthermore, it has been shown that HIV-exposed DCs are dysfunctional in their ability to present antigens and mature, leading to unregulated and persistent inflammation and cytokine production including IFN-α, IL-10, and IL-6. Furthermore, autophagy, which is critical to resolution and control of inflammation, is significantly impaired in dendritic cells infected with HIV [145,166].

### 4.4. NK Cells

Natural killer (NK) cells are lymphocytes that function in many ways as innate immune cells that recognize and eliminate infected or damaged cells [167]. The role of NK cells in atherosclerosis remains uncertain, and whether they possess pro-atherogenic or anti-atherogenic properties warrants further investigation. Single-cell transcriptomic analyses have revealed the heterogeneity of NK cells, suggesting that the balance between inflammatory and anti-inflammatory cells may influence atherosclerosis progression [168]. NK cells might contribute to atherosclerosis by promoting smooth muscle cell apoptosis and necrotic core formation in atherosclerotic plaque [169]. HIV infection is linked to impaired NK cell function, potentially accelerating atherosclerosis in PLWH. Preliminary data indicate that specific NK cell subsets in adolescents with perinatally acquired HIV infection may facilitate oxLDL uptake by vascular macrophages, contributing to atherosclerosis [170].

### 4.5. Platelets

Platelets play a central role in atherosclerosis [143,171,172]. HIV infection appears to heighten platelet activation and interactions with other components of the immune system, ultimately potentiating atherosclerosis [173,174]. Markers of platelet activation, cell adhesion, and signaling molecules which play central roles in leukocyte activation and activity (P-selectin and CD40 ligand) have been found to be elevated on platelets in HIV infection [173]. These activated platelets also have an increased propensity to form platelet–monocyte complexes, which are elevated in settings of MI [173,175]. During ART suppression, markers of platelet activation such as thromboxane2 synthesis and P-selectin remain elevated compared to healthy controls [176]. Recent studies suggest HIV-induced mitochondrial dysfunction as a potential driver of platelet dysfunction and apoptosis [177]. Additionally, thrombogenicity is noted to be significantly higher in PLWH compared to PWoH [178]. Lastly, some ARTs including ritonavir and abacavir have been associated with platelet hyperactivity, whereas raltegravir and rilpivirine appear to have antithrombotic effects on platelet activation, aggregation, and function [178,179,180,181].

## 5. Adaptive Immune System

The adaptive immune system plays a crucial role in the pathogenesis of ASCVD in PLWH (Figure 2). This system is responsible for recognizing and responding to specific antigens, including those present in atherosclerotic plaque. While complex, the adaptive immune response mainly involves antigen-driven activation and differentiation of T and B lymphocytes, as well as the production of antibodies and cytokines.

### 5.1. T Cell Lymphocytes

T cells, including CD4^+^ and CD8^+^ subsets, are essential in the adaptive immune response against HIV. Chronic immune activation and inflammation can lead to T-cell dysfunction and exhaustion, contributing to atherosclerosis [182,183]. Higher frequencies of activated CD4^+^ and CD8^+^ T cells are observed in untreated PLWH with high viral loads compared to those on ART [184,185].

Persistent T cell activation, despite ART, is pivotal in HIV progression. While ART reduces T cell activation, levels remain elevated, especially in CD8^+^ T cells [184,186,187]. Elevated levels of activated T cells (CD38^+^HLA-DR^+^) during ART predict CD4^+^ T cell recovery and mortality risk [188]. The role of T cell activation in ASCVD in PLWH remains debated, with studies showing conflicting associations between activated T cells and ASCVD events and subclinical disease [77,189,190]. Kaplan et al. noted a link between activated CD4^+^ T cells and increased carotid plaque risk in women from the Women’s Interagency HIV Study (WIHS) cohort [190]. Further research is needed to clarify these findings.

#### 5.1.1. CD4^+^ T cells

CD4^+^ T cells play diverse roles in atherosclerosis. Low CD4^+^ counts in PLWH are associated with higher CVD risk and carotid lesions, independent of traditional CVD risk factors [191,192,193,194]. CD4^+^ T cells produce pro-inflammatory cytokines like IFN-γ and IL-17, contributing to endothelial dysfunction and atherosclerosis [82,195,196]. Naïve CD4^+^ T cells can differentiate into subtypes (including Th1, Th2, Th17, Treg), each with distinct effects on atherosclerosis [197,198]. Th1 cells have pro-inflammatory effects, while Th2, Th9, and Th17 effects are controversial [199]. Elevated Th17 cells and IL-17a levels are linked to endothelial dysfunction and CVD in PLWH [82,196]. Conversely, ART-treated PLWH with coronary plaques showed lower Th17 frequencies than those without plaques. Notably, Th17 depletion is a major cause of damage to the gut mucosal barrier, which leads to microbial translocation-associated systemic immune activation, which may explain this connection [200].

#### 5.1.2. Regulatory T Cells (Tregs)

Tregs (CD4^+^CD25^+^FoxP3^+^) generally exhibit anti-inflammatory properties. In PLWH with coronary artery disease, Treg frequencies are elevated, but the cells are less differentiated and express lower levels of atheroprotective markers (CD39/CD73), potentially increasing atherosclerosis risk [199,201,202]. These Tregs also have a distinctive migratory capacity towards atherosclerotic plaques, possibly contributing to decreased plaque stability [202].

#### 5.1.3. Cytotoxic CD4^+^ T cells

Cytotoxic CD4^+^ T cells express markers which have been implicated in atherosclerosis in PLWH, including CD57 (a marker of senescence), CX3CR1 (a chemokine receptor that homes cells to inflamed endothelium), and GPR56 (an adhesion g-coupled protein receptor that is expressed on exhausted T cells) [98,203,204,205]. These cells are elevated in PLWH with subclinical atherosclerosis and often overlap with senescent TEMRA (T effector re-expressing CD45RA) cells (CD28^null^ and CD45RA^+^), which are pro-atherosclerotic [196,206,207,208,209,210,211].

#### 5.1.4. CD8^+^ T cells

CD8^+^ T cells are also crucial in the pathogenesis of atherosclerosis. Expanded effector CD8^+^ T cells in ART-suppressed PLWH are linked to cardiovascular morbidity, with many cells expressing the CX3CR1 receptor [92,93,212,213]. CX3CR1 and its ligand CX3CL1, also elevated in PLWH, are associated with CVD morbidity and predict plaque rupture [214,215,216,217]. CD8^+^ T cells co-localize with CD68^+^ myeloid cells at sites of endothelial dysfunction in simian immunodeficiency virus (SIV)- and simian-human immunodeficiency virus (SHIV)-infected macaques [218]. Atherosclerotic plaques in PLWH are enriched in activated CD8^+^ T cells, which are potent cytokine producers and can promote monocyte procoagulant activity via TNF [92,93,219,220]. CX3CL1 expression by dysfunctional endothelium may attract CX3CR1^+^CD8^+^ T cells, influencing atherosclerosis progression in both PLWH and PWoH [218,220]. However, CD8^+^ T cells can reduce advanced atherosclerosis by limiting increases in Th1 cells and macrophages [221].

### 5.2. B Cells

While the role of B cells in atherosclerosis is well-studied in PWoH [142,222,223], their role in PLWH remains poorly understood [224]. B cells produce antibodies, which play a critical role in the humoral immune response [225]. HIV infection leads to impaired B cell function [226,227], potentially accelerating atherosclerosis in PLWH [224]. B cells are classified into B1 and B2 subsets, the roles of which have primarily been studied in murine models [228]. While B2 cells are the most well-studied subset of B cells, recent research has shown that B1 cells also play a critical role in atherosclerosis [229].

#### 5.2.1. B1 Cells

B1 cells produce natural antibodies, mainly of the IgM isotype, without prior antigen exposure [223]. These antibodies recognize and bind to various antigens including oxLDL and play a protective role in atherosclerosis by promoting clearance of oxLDL from circulation [142,223,230]. B1 cells have not been studied in PLWH.

#### 5.2.2. B2 Cells

B2 cells produce antibodies in response to specific antigens and participate in immune responses to pathogens. In atherosclerosis, B2 cells generate antibodies against oxLDL, forming immune complexes and activating pro-inflammatory pathways, contributing to atherosclerosis [231,232,233]. Antibodies against oxLDL and other plaque antigens may also help clear these antigens and prevent foam cell formation, conferring protection against atherosclerosis [234].

#### 5.2.3. Immune Regulation

B cells also regulate immune responses through interactions with T cells. The cytokine APRIL (a proliferation-inducing ligand) enhances B cell activation and antibody production in HIV [235]. However, increased APRIL levels are associated with atherosclerosis in PLWH [141]. Another B cell-activating cytokine, B-cell activating factor (BAFF), is implicated in pathogenesis of atherosclerosis in HIV infection [236]. Elevated BAFF levels correlate with higher cardiovascular disease risk in PLWH [141]. Targeting B cell function and cytokine production may have therapeutic potential for preventing and treating atherosclerosis in PLWH [224].

## 6. Co-Pathogens

### 6.1. Herpesviruses: CMV, EBV, HSV, HHV-8, VZV

**Figure 3:**
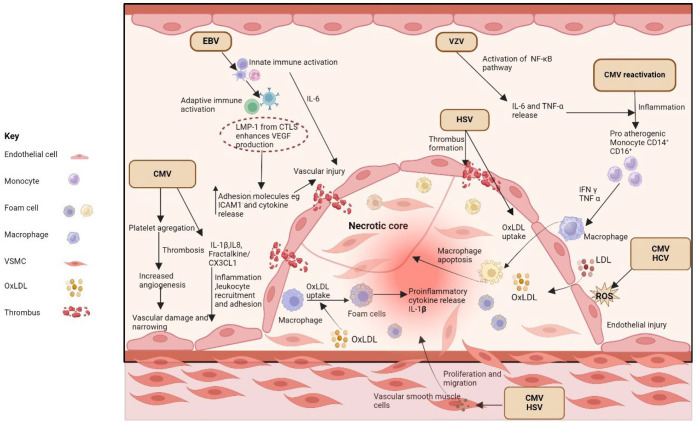
Multiple chronic viral infections are linked to atherosclerosis. Specific viruses such as CMV, HSV-2, and VZV have been associated with subclinical atherosclerosis in PLWH. CMV infection contributes to atherosclerosis through activation of endothelial cells, leading to chronic inflammation and endothelial dysfunction. HCV promotes chronic inflammation, immune activation, and direct vascular injury. HCV and CMV promote LDL oxidation. HSV-2 and HHV-8 can induce thrombogenic and atherogenic alterations in cellular structure, contributing to atherosclerosis. VZV infection may induce chronic inflammation and endothelial dysfunction, contributing to atherosclerosis. EBV’s role in HIV-related atherosclerosis remains controversial, with complex and contradictory associations. Abbreviations: CMV= cytomegalovirus; CTL = cytotoxic T lymphocyte; CX3CL1 = chemokine (C-X3-C motif) ligand 1; EBV= Epstein Barr virus; EC = endothelial cell; HCV= hepatitis C virus; HHV-8= human herpesvirus-8; HSV-2= herpes simplex virus-2; LMP-1 = latent membrane protein 1; NF-κb = nuclear factor kappa B; VEGF = vascular endothelial growth factor.

Several chronic pathogens prevalent in PLWH are thought to contribute to chronic systemic inflammation (Figure 3). The human herpesviridae family, which includes CMV, HSV-2, and VZV, causes chronic infections which are highly prevalent among PLWH. These viruses have been linked to the development of atherosclerosis [237]. Cross-sectional studies have associated CMV, HSV-2, and VZV with subclinical atherosclerosis in PLWH [189,238,239].

#### 6.1.1. CMV

CMV seropositivity, which is more prevalent among PLWH than PWoH, is linked to an increased risk of ASCVD events and the expansion of potentially pro-atherosclerotic T cell subsets [208]. In a cohort of PLWH, CMV seropositivity was associated with an increased risk of ASCVD events, independent of traditional cardiovascular risk factors [98]. CMV is considered a non-traditional risk factor for atherogenesis, contributing to atherosclerosis by activating endothelial cells, an initial and critical step in the process [240]. HIV and CMV infections both have independent associations with inflammation, inflammation-related morbidities, and CVD risk, particularly in the elderly [15]. CMV infection impairs the nitric oxide synthase pathway, leading to endothelial dysfunction and atherosclerosis [241]. In immunocompetent hosts, CMV infection leads to expansion of pathogenic cytotoxic CMV-specific CD4^+^ T cells [203,206] and CD8^+^ T cells [189]. These cells are independently associated with increased cIMT in PLWH [189]. In individuals with CMV, the proportion of CMV-specific cells can constitute up to 30% of the total CD8^+^ T cell count [242]. While CMV DNA has been detected in atherosclerotic plaques in the general population [237,243,244], only a few studies have demonstrated this in PLWH.

In a small study of PLWH, valganciclovir was found to be effective in suppressing CMV DNA and reducing CD8 T cell activation, as indicated by decreased percentages of cells expressing CD38^+^HLA-DR^+^ [245]. This suggests that CMV replication contributes to immune activation in co-infected individuals and that pharmacological suppression of CMV may help reverse this process. However, it remains unclear whether persistent CMV suppression could have clinical benefits in terms of reducing the risk of ASCVD in PLWH. CMV-specific CD4^+^ T cells expressing CX3CR1 are linked to atherosclerosis, and treatments targeting CMV may impact CVD outcomes [204,246,247]. CMV treatment with letermovir in PLWH was found to affect multiple inflammatory biomarkers; however, effects of CMV suppression on long-term ASCVD outcomes have yet to be fully elucidated [248].

#### 6.1.2. Epstein Barr Virus (EBV)

The role of EBV in HIV-related atherosclerosis remains controversial and complex, with limited research conducted in PLWH. While EBV DNA has been detected in atherosclerotic plaques in the general population [237,244], some studies have found no evidence linking EBV infection markers to coronary artery disease or endothelial dysfunction [249,250]. However, EBV infection can increase acute phase proteins, supporting the hypothesis of an indirect effect [249].

In PLWH with acute HIV infection, up to 80–90% of CD8^+^ T-lymphocytes are activated, including not only HIV-specific cells but also those specific to CMV and EBV [251]. This suggests a heightened immune response towards multiple pathogens, potentially contributing to the complex interactions between EBV and atherosclerosis in the context of HIV.

#### 6.1.3. HSV-2 and Kaposi-Sarcoma-Associated Herpesvirus (HHV-8)

Herpesviruses including HSV-2 can induce thrombogenic and atherogenic alterations in the cellular structure of their host [252]. Kaposi-sarcoma-associated herpesvirus (HHV-8) targets the lymphatic and vascular systems and can induce atherogenesis. HHV-8-infected vascular endothelial cells can induce the expression of growth factors that cause angiogenesis, endothelial cell proliferation, enhanced vascular permeability, and cytokine production, potentially contributing to atherogenesis [253].

Co-infection with HHV-8 has been associated with increased inflammation and immune activation in virologically suppressed PLWH [254,255]. Lidón et al. found that HHV-8/HSV-2 co-infection was associated with the progression of subclinical atherosclerosis, measured by cIMT. However, when assessing the independent role of each virus, only HHV-8 had a significant relationship with cIMT progression. Participants co-infected with HHV-8 also had higher levels of inflammation measured with hs-CRP, which was associated with faster cIMT progression [255].

In a separate study among men who have sex with men (MSM) in the Multicenter AIDS Cohort Study, HSV-2 was independently associated with subclinical atherosclerosis. The study also found that that the presence of multiple herpesviruses may increase the risk of coronary artery calcium in MSM living with HIV [238]. These findings support the role of herpesviruses in the pathogenesis of atherosclerosis and highlight the importance of controlling herpesvirus infections in individuals at risk for CVD.

#### 6.1.4. VZV

In the general population, several studies have demonstrated a possible correlation between herpes zoster and the incidence of ischemic cardiac and cerebral events, particularly within the first three months following reactivation [256,257,258]. Limited research has been conducted on the association between VZV and atherosclerosis in PLWH, despite the high frequency of VZV reactivation in PLWH both with and without significant CD4^+^ cell depletion [259,260,261]. In a cohort study of PLWH, VZV seropositivity was associated with an increased risk of ASCVD events, including MI and stroke [262]. Similarly, a study conducted by Masiá et al. investigated the relationship between different types of Herpesviridae and subclinical atherosclerosis by measuring cIMT and flow-mediated dilation (FMD) and found that PLWH with a higher serological response to VZV were more likely to have subclinical atherosclerosis, even after adjusting for the serological response to CMV [239].

The mechanisms by which VZV infection contributes to atherosclerosis are not fully understood, but several potential mechanisms have been proposed. VZV infection has been shown to activate the NF-κB pathway, leading to the production of pro-inflammatory cytokines such as IL-6 and TNF-α [239,263]. These cytokines can recruit immune cells to the arterial wall and promote plaque formation. VZV infection has also been shown to induce endothelial dysfunction, characterized by impaired vasodilation, increased permeability, and elevated expression of adhesion molecules [264]. As VZV is the only herpesvirus with a preventive vaccine, its role in atherosclerosis may be lessened in vaccinated PLWH, especially those with intact immune systems.

### 6.2. HCV

HCV is a common co-infection with HIV and currently affects an estimated 2.5% of the global population [265]. HCV lifecycle is closely related to lipid metabolism in persons with HCV, using triglyceride-rich very-low-density lipoproteins to create infective lipo-viro particles leading to dyslipidemia and hypolipidemia [266]. Chronic HCV has been associated with clinically significant inflammation, increased risk of ASCVD, and clinical consequences of atherosclerosis, including cerebrovascular accidents and overall cardiovascular mortality [267,268]. Moreover, treatment of chronic HCV infection improves mortality compared to those who remain untreated [269]. HCV infection alone promotes expression of atherogenic adhesive molecules soluble ICAM-1, vascular adhesion molecule-1 (VCAM-1), and E-selectin, as well as pro-inflammatory cytokines associated with CVD mortality like TNF-α [270,271,272,273].

Co-infection with HCV is a known risk factor for CVD in PLWH that, like HIV, is not accounted for in clinical calculators of ASCVD risk. HIV and HCV co-infection poses nearly twice the risk of coronary heart disease compared to HIV infection alone [274,275]. Compared to PWoH, co-infected PLWH had higher levels of many pro-inflammatory cytokines and chemokines including IL-1α, IL-1β, IL-6, IL-12p40, IL-12p70, and TNF-α [276].

HCV/HIV co-infection is also associated with lower levels of LDL but paradoxically higher levels of proprotein convertase subtilisin kexin 9 (PCSK-9) [277], a protein which decreases LDL receptor expression and thus increases circulating LDL concentrations, and likely has other pro-inflammatory and atherogenic roles in uninfected individuals [278]. PCSK-9 levels may be related to inflammatory mechanisms such as IL-6-mediated pathways, given higher levels of IL-6 measured in untreated co-infected PLWH compared with PLWH without HCV [277]. Treatment of HCV with direct-acting antivirals (DAA) has been reported to normalize levels of PCSK9 in co-infected PLWH back to those of PLWH without HCV, as measured about 43 weeks after end of treatment [278].

HCV eradication with DAA, with or without ribavirin-IFN, in HIV/HCV co-infected individuals reduced some other biomarkers of inflammation such as sE-selectin and sCD163, but other markers of inflammation including IL-6, IL-8, sCD14, sVCAM-1, and sICAM-1 and overall LDL levels were generally not affected by therapy [278,279]. Some studies of ribavirin-IFN therapy without DAA in HIV/HCV co-infected individuals have shown reduction in some inflammatory markers, such as sCD163 and sICAM-1 as well as oxLDL, at 24 weeks after treatment [280]. Notably, Chew et al. found that this difference was largely driven by hepatic inflammation, as it was largely attenuated by adjusting for ALT levels [268]. Much more research is needed to investigate the mechanisms driving the increased risk of atherosclerotic vascular disease in PLWH co-infected with HCV.

## 7. Summary and Future Directions

The inflammatory and immune mechanisms driving the ASCVD risk in HIV infection are multi-dimensional. First, there are numerous clinical and environmental factors that induce inflammation in PLWH, such as changes in the microbiota and lipid derangements, along with convergence with the substance use epidemic (Figure 1). Overall, there is a generalized state of chronic inflammation with elevated levels of key cytokines and chemokines (IL-6, TNF-α, IL-8, CCR5, CCR2, CX3CR1) and immune activation and dysfunction among numerous immune cell types of the innate and adaptive immune systems that are not functionally restored fully by ART-induced viral suppression (Figure 2). There are immune cell subsets that have been implicated in atherosclerosis but remain understudied in HIV infection and should be actively pursued (dendritic cells, NK cells, B cells). Furthermore, PLWH are often co-infected with co-pathogens that have been reported to promote atherogenic pathways that may be accentuated and accelerated with concomitant viral infections (Figure 3). This represents a much-needed area for future investigations, given the high prevalence of co-infections with CMV and/or HCV and HIV, thereby representing a significant public health burden globally. There is only a limited number of available therapeutic immunomodulators for the treatment of atherosclerosis, regardless of HIV status, though several promising targets exist that could be tested (Table 1). There is an urgent need to investigate immunomodulatory pathways that could be therapeutic targets in HIV-associated ASCVD, and to test these in well-designed clinical trials, given the disproportionately high burden of atherosclerosis in this population.

## Figures and Tables

**Table 1:** Current and novel therapeutic interventions targeting the immune system for ASCVD prevention and MACE reduction.

Therapeutic Interventions Targeting the Immune System That Have Been Assessed for ASCVD Prevention and MACE Reduction					
Therapeutic Intervention	Target Cytokine/Cell Line/Inflammatory Marker	Mechanism of Action	Evidence in Persons without HIV	Experience in Persons with HIV	References
Canakinumab	IL-1β	Monoclonal antibody neutralizes IL-1β signaling	In the CANTOS trial, interleukin-1β blockade with canakinumab at a dose of 150 mg every 3 months led to a significantly lower rate of recurrent CVD events than placebo, independent of lipid-level lowering.	8 weeks of canakinumab treatment significantly lowered inflammatory biomarkers, including hs-CRP and sCD163. Additionally, leukopoiesis as measured by bone marrow FDG-PET signal and arterial inflammation decreased.	[100,101]
Tocilizumab	IL-6 receptor (IL-6R)	Monoclonal antibody targets and blocks IL-6R	In the ASSAIL-MI randomized control trial (RCT), tocilizumab increased salvage of viable myocardium in persons with acute STEMI.	In a small RCT, tocilizumab was found to be safe and decreased IL-6, but significantly increased cholesterol levels.	[102,103]
Methotrexate (MTX)	IL-1β, IL-6, CRP	Modulates inflammation by promoting adenosine uptake and inhibiting transmethylation reactions, subsequently reducing inflammatory biomarkers like CRP, IL-6, and TNF-α	In the CIRT trial, among persons with stable atherosclerosis, low-dose MTX did not reduce levels of IL-1β, IL-6, or CRP and did not result in fewer CVD events than placebo.	In a small study of PLWH with increased ASCVD risk, low-dose MTX had more adverse safety events than placebo. Additionally, low-dose MTX had no significant effect on endothelial function or inflammatory markers, but was associated with a significant decrease in CD8^+^ T cells.	[104,105]
Colchicine	IL-8, NLR3 inflammasome related cytokines (e.g., IL1-β)	Diminishes neutrophil inflammatory function and migration	In the COLCOT and LoDOCo2 trials, colchicine led to a significantly lower risk of ischemic CVD events than placebo. In the COPS Trial, no significant reductions in acute coronary syndromes or non-cardiometabolic stroke were observed, indicating that effects may be dependent on specific study population.	A small study examining colchicine’s effect on coronary endothelial function and serum inflammatory markers in PLWH without coronary artery disease (CAD) did not show improvement on endothelial function or inflammatory markers.	[106,107,108,109,110,111]
Statins	HMG-CoA reductase enzyme	Multiple: inhibits cholesterol synthesis in liver; inhibits NF-κB, an important transcription regulatory protein in inflammatory response; activates NOS gene transcription to stimulate production of nitric oxide	United States Preventive Services Task Force (USPSTF) recommends statins for primary prevention of ASCVD in adults aged 40 to 75 years with a 10-year ASCVD risk of 10% or greater and a nuanced approach among those with 10-year ASCVD risk of 7.5% to 10% based on multiple randomized trials and meta-analyses of cohort studies demonstrating benefit in reducing ASCVD.	REPRIEVE trial found that pitavastatin reduced risk of MACE by 35% compared to placebo in PLWH with low-moderate ASCVD risk. The REPRIEVE mechanistic sub-study demonstrated reduction in markers of vascular inflammation in those who received statin. Similar findings have been reported in smaller observational studies like INTREPID, SATURN-HIV, and ACTG A5255.	[80,112,113,114,115,116]
PCSK9 inhibitors	LDL receptors	Binds to PCSK9 protein preventing its interaction with LDL receptors, and stops their degradation, allowing more uptake of cholesterol from the blood into the liver	PCSK9 inhibition with evolocumab with concurrent statin therapy lowered LDL cholesterol levels by an additional 15–20% and reduced the risk of cardiovascular events.	In a small study, evocolumab reversed coronary artery dysfunction, but had no effect on reducing markers.	[117,118]
Aspirin	Platelets	Cyclo-oxygenase-1 pathway	Based on multiple clinical studies, USPSTF guidelines recommend aspirin use for the primary prevention of CVD events in adults aged 40 to 59 years who have a 10% or greater 10-year CVD risk, with moderate certainty; has a small net benefit.	Aspirin treatment for 12 weeks had no major impact on sCD14, IL-6, sCD163, D-dimer, T-cell or monocyte activation, or flow-mediated dilation.	[119]
Dipyridamole	Platelets	Inhibits adenosine re-uptake	Older anti-platelet agent not currently recommended for primary prevention of ASCVD.	In a small RCT, dipyridamole increased extracellular adenosine levels and decreased T-cell activation significantly among PLWH virally suppressed on ART.	[120]
**Novel Therapeutic Strategies Targeting the Immune System to Reduce ASCVD and MACE**					
**Therapeutic Intervention**	**Target Cytokine/Cell Line/Inflammatory Marker**	**Mechanism of Action**	**Evidence in Persons without HIV**	**Experience in Persons with HIV**	**References**
IL-17a Blockade	IL-17a	Inhibit effect of IL-17a on monocyte and macrophage function, chemokine expression, and leukocyte migration	Functional blockade of IL-17a in vitro has been shown to prevent atherosclerotic lesion progression and promote plaque stabilization in murine models of ASCVD. Clinical studies of persons treated for psoriasis with IL-17a antagonists have shown reduction in early indicators of CVD.	Not widely studied.	[121,122,123]
IL-18 antagonists	IL-18	Inhibit effects of IL-18 on monocyte and macrophage activation, thus reducing production of interferon gamma and enhancing Th1 responses	Pro-atherogenic effects of IL-18 demonstrated in murine model of atherosclerosis.	HIV-infected macrophages had increased foam cell formation, expression of NLRP3 inflammasome components, and production of downstream cytokines including IL-18.	[86,87,124]
Maraviroc	CCR5 chemokine receptor	CCR5 receptor antagonist	Not studied for ASCVD prevention or MACE reduction.	In non-human primate studies of simian immunodeficiency virus (SIV), maraviroc led to fewer activated CD163+ macrophages in the heart and preserved diastolic function. ART intensification studies with maraviroc showed decreased expression of vascular cellular adhesion molecule-1 (VCAM-1) compared to controls.	[125,126]
Rilpivirine	Platelet activation markers (P-selectin, CD40 ligand)	Inhibits thrombin and ADP-triggered platelet aggregation, degranulation, and activation	Not studied for ASCVD prevention or MACE reduction.	Rilpivirine was found to prevent the progression of thrombotic CVD by inhibiting thrombin and ADP-triggered platelet aggregation, degranulation, and activation without increasing risk of bleeding in murine models of ASCVD.	[127]
Janus kinase (JAK) inhibitors	JAK-STAT signaling pathway	Inhibits specific enzymes in the JAK family including JAK1, JAK2, JAK3, and TYK2, which are involved in the signaling pathways that regulate hematopoiesis and immune activation	Tofacitinib has been shown to increase risk of MACE in persons with rheumatoid arthritis (RA) compared to TNF-α antagonists in a RCT. In a smaller study, tofacitinib reduced markers of endothelial dysfunction among persons with RA.	Ruxolitinib has been shown to be safe in a phase 2 study and reduced IL-6 and sCD14 in ART-treated PLWH.	[128,129,130]
HIV vaccines	Dendritic cells (DCs)	Inactivated HIV-1 virus introduced to autologous monocyte-derived DCs, leading to enhanced HIV-1-specific T cell responses	Not studied for ASCVD prevention or MACE reduction.	Potential decrease in HIV-mediated chronic inflammation.	[131,132,133]
Anti-inflammatory nanoparticles and antigenic peptides	Atheroma-specific dendritic dells	Induces an anti-inflammatory milieu facilitated by DCs	Targeted delivery of engineered anti-inflammatory nanoparticles and antigenic peptides to atheroma-specific DCs has been shown to reduce atherosclerotic lesions and induce a more anti-inflammatory milieu.	Not studied.	[134]
Sivelestat	Neutrophil elastase inhibitor	Inhibits neutrophil extracellular trap (NET) formation	Delivering sivelestat utilizing a plaque-targeting, neutrophil-hitchhiking liposome (cRGD-SVT-Lipo) reduced CAD plaque area and increased plaque stabilization in a murine model of ASCVD.	Not studied.	[135]
MCC950	NLRP3 inflammasome	Downregulates NLRP3 pro-inflammatory activity, which is implicated in foam cell formation and pro-inflammatory cytokine production	Selective inhibition of NLRP3 with MCC950 downregulates inflammasome pro-inflammatory activity.	Inhibition of NLRP3 with MCC950 in HIV-infected macrophages reduced foam cell formation in vitro.	[87]
Apolipoprotein A-I mimetic peptides	Macrophages	Reduces biomarkers of macrophage activation	Small and short-term early stage trials, mostly based on imaging endpoints, have shown a favorable effect of rapidly reducing atherosclerotic plaque volume. However, larger clinical studies have failed to show significant benefit.	Apolipoprotein A-I mimetic peptides reduced biomarkers of macrophage activation in a murine model of treated HIV.	[136,137]
Methylglyoxal-bis-guanylhydrazone (MGBG)	Macrophages	Inhibits biosynthesis of polyamines, which are necessary for macrophage activation, proliferation and differentiation	Not extensively studied for ASCVD prevention or MACE reduction.	In a nonhuman primate model of SIV, MGBG has been demonstrated to reduce cardiovascular inflammation, carotid artery intima-media thickness, and fibrosis.	[138]
Natalizumab	α4 integrin	Monoclonal antibody against α4 integrin decreases macrophage traffic to cardiac tissue	Persons receiving natalizumab for treatment of multiple sclerosis have beneficial changes in lipid profiles and antioxidant uric acid levels.	Decreased trafficking of macrophages to cardiac tissue and decreased cardiac pathology in nonhuman primate model of SIV.	[139,140]
B cell activation factors	APRIL (a proliferation-inducing ligand), BAFF (B-cell activating factor)	Reduces B cell activation and associated cytokines	Not extensively studied for ASCVD prevention or MACE reduction.	Increased levels of APRIL have been associated with the development of atherosclerosis. Increased levels of BAFF are associated with increased risk of ASCVD.	[141,142]

ART = antiretroviral therapy; ASCVD = atherosclerotic cardiovascular disease; BAFF = B-cell activating factor; CD = cluster of differentiation; COX = cyclooxygenase; CVD = cardiovascular disease; DC = dendritic cells; FDG-PET = 18-fluoro-deoxyglucose positron emission tomography; HIV = human immunodeficiency virus; HMG-CoA = β-hydroxy β-methylglutamyl coenzyme A; hs-CRP = high-sensitivity C-reactive protein; JAK-STAT = janus-kinase/signal transducers and activators of transcription; LDL = low-density lipoprotein; MACE = major adverse cardiovascular events; MTX = methotrexate; NET = neutrophil extracellular traps; NF-kappa β = nuclear factor kappa-beta; NLRP3 = nucleotide-binding domain, leucine-rich-containing family pyrin domain-containing 3; NOS = nitric oxide synthetase; PBMCs = peripheral blood mononuclear cells; PCSK9 = proprotein convertase subtilisin/kexin type 9; PLWH = persons living with HIV; RA = rheumatoid arthritis; RCT = randomized controlled trial; sCD = soluble cluster of differentiation; SIV = simian immunodeficiency virus; TNF = tumor necrosis factor; USPSTF = United States Preventive Services Task Force; VCAM-1 = vascular adhesion molecule-1.

## Data Availability

Data sharing is not applicable.
